# An Omalizumab Biobetter Antibody With Improved Stability and Efficacy for the Treatment of Allergic Diseases

**DOI:** 10.3389/fimmu.2020.596908

**Published:** 2020-11-27

**Authors:** Peipei Liu, Zhongzong Pan, Chunyin Gu, Xiaodan Cao, Xiaowu Liu, Jianjian Zhang, Zheng Xiao, Xueping Wang, Haibing Guo, Dianwen Ju, Su-Jun Deng

**Affiliations:** ^1^Department of Biological Medicines & Shanghai Engineering Research Center of Immunotherapeutics, Fudan University School of Pharmacy, Shanghai, China; ^2^Shanghai Jemincare Pharmaceuticals Co., Ltd., Shanghai, China

**Keywords:** IgE, allergic diseases, biobetter, affinity, stability, half-life, efficacy

## Abstract

The critical role of IgE in allergic diseases is well-documented and clinically proven. Omalizumab, a humanized anti-IgE antibody, was the first approved antibody for the treatment of allergic diseases. Nevertheless, omalizumab still has some limitations, such as product instability and dosage restriction in clinical application. In this study, we attempted to develop an omalizumab biobetter antibody with the potential to overcome its limitations. We removed two aspartic acid isomerization hotspots in CDRs of omalizumab to improve antibody candidate’s stability. Meanwhile, several murine amino acids in the framework region of omalizumab were replaced with human source to reduce the potential immunogenicity. Yeast display technology was then applied to screen antibody candidates with high binding affinity to IgE. Moreover, YTE mutation in Fc fragment was introduced into the candidates for extending their serum half-life. A lead candidate, AB1904Am15, was screened out, which showed desired biophysical properties and improved stability, high binding affinity and elevated potency *in vitro*, prolonged half-life in human FcRn transgenic mouse, and enhanced *in vivo* efficacy in cynomolgus monkey asthma model. Overall, our study developed a biobetter antibody of omalizumab, AB1904Am15, which has the potential to show improved clinical benefit in the treatment of allergic diseases.

## Introduction

Allergic diseases, such as allergic asthma, atopic dermatitis, allergic rhinitis and food allergy, represent a chronic disorder of the immune system to various environmental allergens, for example, house dust mites, pollens and animal dander. Moreover, the incidence of allergic diseases is rising all over the world (1, 2). Among them, asthma is a common global chronic respiratory disease which influences over 300 million people, and its prevalence is rising around the world (3, 4). Asthma markedly reduces the quality of life, causes hospitalizations and wreaks significant social and economic consequences (5).

IgE plays a critical role in the pathophysiology of allergic disease (2, 6–9). It mediates an allergic reaction *via* interaction with its two receptors, namely the high-affinity receptor FcϵRI and the low-affinity receptor CD23 for IgE (10). FcϵRI is expressed on basophils and mast cells, and sensitizes these cells to respond to allergens (11). CD23 is expressed on B cells, and plays a role in IgE-mediated antigen presentation and the feedback regulation of IgE antibody production (12). On exposure to specific antigens, the allergens bind to IgE and crosslink the IgE/FcϵRI complexes, subsequently trigger the release of inflammatory mediators, and then, induce various allergic symptoms (13, 14). IgE has been an ideal target for drug development because of its essential role in mediating allergic response in human (6, 7).

Omalizumab (rhuMab E25; Xolair) is a humanized IgG1 antibody containing approximately 5% murine and 95% human sequences. It is the first and the only anti-IgE monoclonal antibody approved in several countries for the treatment of severe or moderate-to-severe persistent allergic asthma (15, 16). Furthermore, it has also been approved to treat patients with recalcitrant, antihistamine-resistant chronic idiopathic urticaria (17, 18). Omalizumab has exhibited robust clinical efficacy, and showed potential to treat a wide range of other allergic diseases (19–22). It binds to the constant region of free IgE and prevents free IgE binding to IgE receptors (23, 24). The reduction of serum free IgE levels results in downregulation of FcϵRI expression on effector cells (24, 25), further dampening the effector cell response to allergens (26). Besides, it also reduces surface levels of IgE on FcϵRI expressing cells (27). The decline of IgE surface levels on FcϵRI expressing cells results in the losing of their ability to bind allergens and to undertake IgE-dependent activation.

Despite showing efficacy against allergic diseases, in particular against allergic asthma, omalizumab still has some limitations (28–31). Firstly, there are two typical isomerization hotspots of aspartic acid-glycine (Asp-Gly) sequences at residues 32–33 in complementarity-determining regions (CDR) of light chain and at residues 55–56 in CDR of heavy chain, which resulted in chemical instability and lower affinity to IgE (28, 29, 32). The variants deriving from Asp isomerization reaction also bring challenges to the drugs manufacture, the consideration of efficacy and safety as well as the shelf life of products (33, 34). Therefore, there exists an opportunity for the generation of an optimized version of omalizumab, which not only avoids “deactivating” event of Asp isomerization, but also displays equal to or greater affinity to IgE than omalizumab. Secondly, some murine amino acids are still in the framework region of omalizumab, which might raise the potential immunogenicity risks due to generating human anti-mouse antibody response (35, 36). Thirdly, the dosage of omalizumab for clinical treatment is restricted to human body weight and the total free IgE level (30). Some patients whose body weight or free IgE level that deviate from a qualified range may be excluded from this treatment or required multiple injections or higher doses (27). In addition, potential disadvantages of continuous and potentially life-long administration of high doses of omalizumab may sustain the high risk of anaphylactic or serum-sickness-like reactions (37, 38). Therefore, it is necessary to optimize anti-IgE therapeutics for overcoming these limitations associated with omalizumab.

In this study, we sought to develop an omalizumab biobetter antibody candidate with the potential to overcome above mentioned challenges. Firstly, we applied antibody engineering technology to optimize omalizumab sequence to reduce the potential immunogenicity risk, and remove the two typical Asp isomerization hotspots in CDRs to improve stability. Secondly, affinity mature was carried out by yeast surface display platform using above optimized sequence as a template to screen for higher affinity antibody candidates. Finally, the crystallizable fragment (Fc) engineering technology was applied to extend serum half-life of antibody. The biophysical properties, stability profile and biological activity as well as efficacy of candidates were also evaluated.

## Materials and Methods

### Reagents

Omalizumab was purchased from Novartis (Basle, Switzerland). L0H0 was an in-house produced omalizumab biosimilar antibody. MEM medium, FBS, Formic acid and acetonitrile were obtained from Thermo Fisher Scientific (Pittsburg, PA, USA). Iodoacetamide (IAM), dithiothreitol (DTT), urea, β-mercaptoethanol, HRP-labeled streptavidin, guanidine hydrochloride and ammonium carbonate were obtained from Sigma-Aldrich (St. Louis, MO, USA).

### Cell Culture

ExpiCHO-S™ cells were obtained from Thermo Fisher Scientific (Waltham, MA, US) and cultured in ExpiCHO medium according to manufactory’s instruction. Yeast cells were acquired from American Type Culture Collection (Rockville, MD, USA) and cultured according to manufactory’s introduction. RBL-2H3/FcεRI cells were generated by Shanghai GeneChem (Shanghai, China) and cultured in MEM medium supplemented with 15% FBS in a 5% CO_2_ incubator at 37°C.

### Generation of Anti-IgE Antibody Candidates

At first, we selected the most homologous germline antibody genes from IMGT database for humanization of omalizumab to minimize possibility of immunogenicity in human, and removed two Asp isomerization hotspots in CDR-L1 at L32–33 and in CDR-H2 at H55–56 for improving candidate stability. And then, we took this engineered candidate molecule as template, designed mutation library of each CDR in variable domains, displayed Fab on the surface of yeast cells (39) and sorted the affinity matured candidates by flow cytometry (BD Science, CA, USA).

### Antibody Expression and Purification

Candidates were expressed in ExpiCHO-S™ cells. The VH and VL variable regions of antibody candidates were subcloned into expression vectors containing constant regions of IgG1 isotype. The candidates’ heavy chain and light chain expression vectors were co-transfected into ExpiCHO-S™ cells. After 8 days of growth, the supernatant was harvested and passed over AmMag™ Protein A Magnetic Beads(Genscript Biotech, Jiangsu, CN)according to the manufacturer’s instruction. Yields of purified antibody candidates were determined based on their UV absorbance at 280 nm by a Nanodrop instrument (Thermo Fisher Scientific, PA, USA).

### Size Exclusion Chromatography

SEC-HPLC was performed to quantify monomer purity of candidates on a Waters Alliance 2695 HPLC system with TOSOH TSKgel G3000W_XL_ column (300 × 7.8 mm, 5 μm). The mobile phase was 200 mM sodium phosphate (pH 6.8) at the flow rate of 0.5 ml/min. Samples were measured by UV absorbance at the wavelength of 280 nm. Data were analyzed by Waters Empower 3 Enterprise software (Waters, MA, USA).

### Hydrophobic Interaction Chromatography

HIC-HPLC was performed to evaluate hydrophobicity of candidates on a Waters Alliance 2695 HPLC system with Thermo MAbPacHIC-10 column (4 × 250 mm, 5 μm). The mobile phase A consisted of 1 M ammonium sulfate with 50 mM sodium phosphate (pH 7.0), and the mobile phase B consisted of 50 mM sodium phosphate (pH 7.0). Samples were injected and eluted in a linear gradient at the flow rate of 0.8 ml/min, measured by UV absorbance at the wavelength of 214 nm. Data was analyzed by Waters Empower 3 Enterprise software.

### Reduced and Non-Reduced Capillary Electrophoresis-Sodium Dodecyl Sulfate

CE-SDS was conducted on Beckman PA800 Plus Pharmaceutical Analysis system (SCIEX, CA, USA) to separate candidates’ size variants under reduced or non-reduced conditions. For non-reduced condition, samples were diluted by SDS-MW sample buffer and incubated with IAM at 70°C for 10 min. For reduced condition, β-mercaptoethanol was mixed with samples and incubated at 70°C for 10 min. And then, both non-reduced and reduced samples were injected and separated according to the instruction of IgG Purity and Heterogeneity Kit (Beckman Coulter, NJ, USA). Acquired data were processed by 32 Karat software (SCIEX, CA, USA).

### Isoelectric Point and Charge Variants by Imaged Capillary Isoelectric Focusing

Isoelectric point and charge variants were characterized on iCE3 system(ProteinSimple, CA, USA). Samples were mixed with pharmalytes, 1% methyl cellulose, pI marker and ddH_2_O until a final protein concentration of 0.2 mg/ml was reached. Samples were pre-focused at 1,500 V for 1 min and focused at 3,000 V for 10 min with detection at 280 nm. Data were acquired and analyzed by Chrom Perfect software(ProteinSimple, CA, USA).

### Thermal Stability by Differential Scanning Fluorimetry

Protein Thermal Shift™ Dye kit (Thermo Fisher Scientific, PA, USA) was applied to evaluated the melting temperature (Tm) value of candidates. According to manufactory’s instruction, samples were mixed with protein thermal shift™ buffer and protein thermal shift™ dye solution. Protein melt reactions were run on an Applied Biosystems Real-Time PCR System (QuantStudio 3, Thermo Fisher Scientific, USA). Data was collected and transferred to Protein Thermal Shift Software (Version 1.3) to calculate the Tm value of candidates from the melt curve.

### Identification of Post-Translational Modification by Peptide Mapping

For identification of PTMs induced in forced degradation study, samples were assessed by peptide mapping using Waters ACQUITY UPLC H-ClassBio System coupled with Waters Xevo G2-XS QTOF mass spectrometer. Samples were denatured by 6 M guanidine hydrochloride, and reduced and alkylated with 60 mM DTT and 150 mM IAM, respectively. And then, the reduced alkylated samples were desalted and buffer exchanged to 50 mM ammonium carbonate buffer (pH 8.0) for digesting with trypsin (Promega, Madison, WI, USA). The digested samples were injected onto a Waters ACQUITY UPLC BEH300 C18 column (2.1 × 150 mm, 1.7 μm) coupled online to Waters Xevo G2-XS Q-TOF mass system for separation and detection. Data were collected and analyzed by Waters UNIFI Scientific Information System software.

### Stability Evaluation by Forced Degradation Study

A series of stress conditions were applied to evaluate the stability of antibody candidates. Samples were expressed, purified and buffer exchanged into the formulation buffer, which contained 5 mM histidine, 85 mM sucrose, and 0.01% polysorbate 20 (pH 6.0). After putting on the stress conditions for several times, samples were analyzed by SE-HPLC, CE-SDS, and iCIEF for obtaining stability proﬁling. To identify degradation variants, peptide mapping was used to characterize the stressed samples by LC–MS/MS.

### Surface Plasmon Resonance Analysis

The binding affinity of antibody candidates to IgE was evaluated by Biacore 8K (GE Healthcare, MA, USA) instrument. The Biotin CAPture reagent was captured on Sensor Chip CAP (GE Healthcare, MA, USA), followed by capturing 2 μg/ml of biotinylated full length human IgE (Biotin-FL hIgE, Abbiotec, CA, USA) with a flow rate of 10 μl/min for 60 s. Kinetic measurements were executed by injecting serially diluted antibodies with a flow rate of 30 μl/min for 180 s, followed by measuring dissociation for 400 s. The data was globally ﬁtted using a 1:1 binding model. The K_D_ value was evaluated by Biacore Evaluation software (GE Healthcare, MA, USA).

### FcϵRI Binding Inhibition Assay

The ability of antibodies to block human IgE binding to its receptor FcϵRI was measured by a competitive ELISA. The ELISA plate was coated with 0.5 μg/ml recombinant soluble FcϵRI protein (R&D, MN, USA) overnight at 4°C. The plates were blocked with blocking buffer (2% BSA in PBST) at 37°C for 1 h after washing with PBST (PBS/0.05% Tween-20). Biotinylated human IgE was mixed with serially diluted candidates and incubated at 37°C for 2 h, then added to the plate for further incubation at 37°C for 1 h. The plate was washed and HRP-labeled streptavidin was added to detect IgE bound to FcϵRI. After incubation for 1 h, the plate was washed and TMB solution (Biopanda, Belfast, UK) was added to detect the optical density at 450 nm. Data was analyzed by GraphPad Prism (Version 8).

### Histamine Releasing Assay by FcϵRI-Expressing RBL-2H3 Cells

FcϵRI-expressing RBL-2H3 cells were cultured with serially diluted antibodies and human IgE (Abbiotec, CA, USA) to a final IgE concentration of 1.2 μg/ml at 37°C, 5% CO_2_ overnight. The cells were washed and the 1 μg/ml of polyclonal anti-human IgE (R&D, MN, USA) was added to the cells, following by incubation at 37°C for 30 min. The supernatant was collected and the histamine concentration was determined by Histamine Dynamic kit (CisBio, Codolet, France) according to the manufacturer’s instruction.

### Pharmacokinetic Study in Human FcRn Transgenic Mouse

A pharmacokinetic study was conducted in human FcRn (hFcRn) transgenic mice and the study protocol was approved by Institutional Animal Care and Use Committee (IACUC). Human FcRn transgenic mice were purchased from Biocytogen (Beijing, CN), 6–8 weeks old male mice were used. Mice received a single dose of 10 mg/kg test antibody *via* subcutaneous administration (n = 4/group). Serum samples were collected from submandibular vein by cheek puncture at the following time points: pre-dose, 2, 6, and 24 h post dose; and 2, 3, 4,7, 10, 14, 21, 28, 35, and 42 days post dose. Serum concentrations of the antibody were measured by an ELISA method. Briefly, anti-human IgG1 (Fc specific, ABCAM, UK) antibody was used as the capture reagent and the mouse anti-human Fab (ABCAM, UK) conjugated to HRP was used for detection. Data was analyzed using GraphPad Prism (Version 8) and pharmacokinetics parameters were determined using Phoenix WinNonlin 6.3 (Certara, NJ, USA).

### *In Vivo* Efficacy Study in a Cynomolgus Monkey Model of Asthma

*In vivo* efficacy study was conducted in a cynomolgus monkey model of asthma and the study protocol was approved by IACUC. Cynomolgus monkeys were purchased from Hainan Lingzhang Experimental Animal Development Co., Ltd (Hainan, China). 3–7 years old male cynomolgus monkeys weighing between 3 and 7 kg were used. To develop the allergic asthma models, monkeys were sensitized with dinitrophenyl-Ascaris suum (DNP-As) allergen following the procedure as previously described (40). After modeling, monkeys received a dose of 10 mg/kg test antibody once a week *via* subcutaneous administration (n = 4/group) for 3 weeks. Humanized IgG1 antibody was used as isotype control. Omalizumab was used as a positive control. Airway resistance (R_L_) and lung dynamic compliance (Cdyn) were measured at 24 h post 1st and 3rd dosing.

### Statistical Analysis

Statistical analysis was performed by two-way analysis of variance (ANOVA) *via* GraphPad Prim 8. The data was expressed as mean ± standard deviations (SD) (*P <0.05, **P <0.01, ***P <0.001).

## Results

### Affinity Maturation by Yeast Surface Display Platform

**Figure 1:**
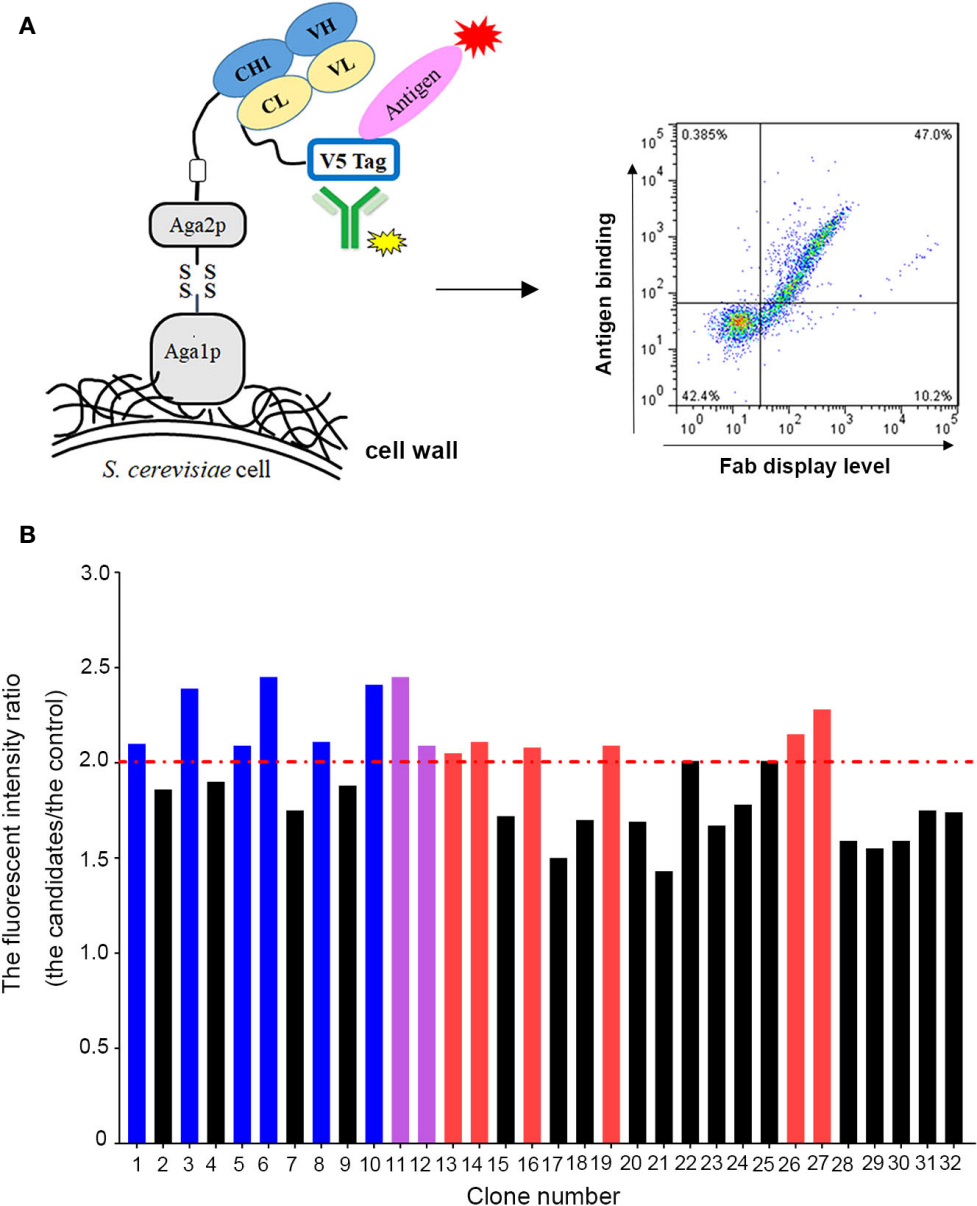
Yeast surface display platform was applied to generate antibody candidates with high affinity to IgE. **(A)** Schematic illustration of application of yeast surface display platform for affinity maturation. **(B)** Screening of anti-IgE antibody candidates with higher affinity to IgE by flow cytometry. The clone, which displayed Fab of omalizumab on the surface of yeast cells, was used as control. The fluorescent intensity ratio (the candidates/the control) higher than 2.0 was considered as positive clones. Red bars represented the clones with low diversity in sequence. Purple bars represented the clones with PTM sites in their CDRs. Blue bars represented the clones were chosen as candidates for further study.

**Figure 2:**
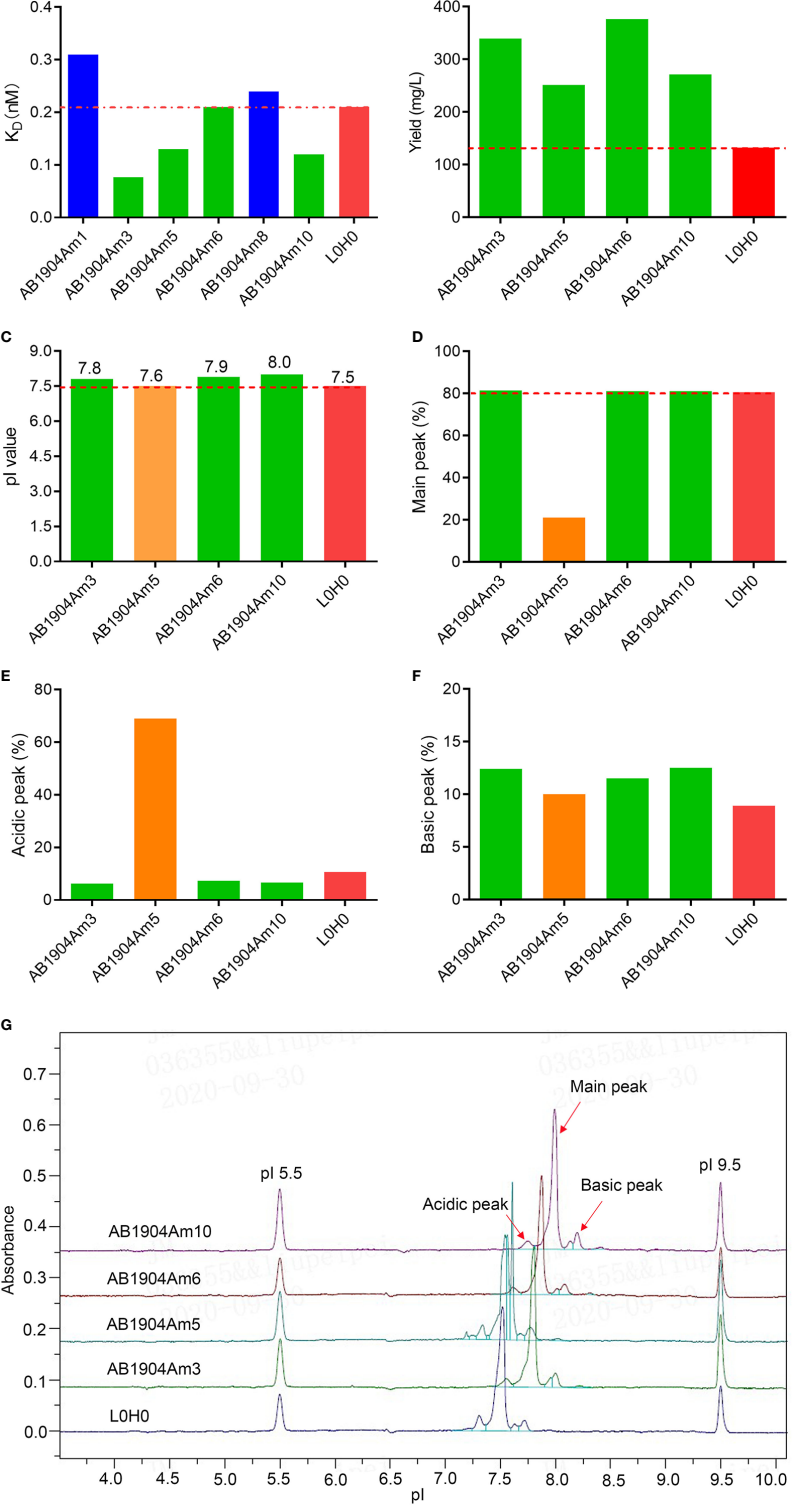
Affinity assessment and biophysical characterization of anti-IgE antibody candidates after affinity maturation. **(A)** Affinity analysis of antibody candidates by SPR. **(B)** Expression level of anti-IgE antibody candidates compared with L0H0. **(C)** The pI values of antibody candidates by iCIEF. **(D–F)** Charge variants analysis of antibody candidates by iCIEF. **(G)** Charge variants profiles of antibody candidates by iCIEF. L0H0, an in-house generated omalizumab biosimilar antibody, was used as control. AB1904Am3, AB1904Am6 and AB1904Am10 were chosen as candidates, and YTE mutation in Fc fragment was introduced into them to yield AB1904Am13, AB1904Am16 and AB1904Am15, respectively, for the following stability study.

To improve the antigen affinity of antibody candidates, yeast surface display technology was used for affinity maturation. At first, we removed two aspartic acid isomerization hotspots in CDR-L1 at L32 and in CDR-H2 at H55 for improving antibody stability of omalizumab. In addition, seven murine amino acids in the framework region of omalizumab were replaced with human source to minimize the possibility of immunogenicity (**Supplementary Figure 1**). And then, we took the engineered antibody sequence as template, designed mutation library of each CDR in variable domains, displayed Fab on the surface of yeast cells. Flow cytometry was used for sorting and screening (**Figure 1A**). As shown in **Figure 1B**, after three rounds of screening, 14 out of 32 positive clones showed higher IgE-specific binding activity by flow cytometry evaluation, that is, the average fluorescence intensity was 2-fold higher compared with the control clone, which displayed Fab of omalizumab on the surface of yeast cells. The clones with high diversity in sequences and no potential post-translational modification (PTM) sites in CDRs were selected. Therefore, Am1, Am3, Am5, Am6, Am8, and Am10 were selected as candidates for affinity evaluation by SPR. As shown in **Figure 2A**, the K_D_ values of AB1904Am3, AB1904Am5 and AB1904Am10 were significantly lower than L0H0, an in-house generated omalizumab biosimilar antibody, while the value of AB1904Am6 was almost the same as L0H0. The results also showed the K_D_ values of AB1904Am1 and AB1904Am8 were higher than L0H0, which indicating the lower affinity of AB1904Am1 and AB1904Am8 to L0H0. Therefore, AB1904Am3, AB1904Am5, AB1904Am6 as well as AB1904Am10, which had higher or similar affinity compared with L0H0, were chosen as candidates for the further evaluation.

### Biophysical Characterization of Anti-IgE Antibody Candidates

The discovery and development challenges of therapeutic antibodies are often associated with the inherent biophysical properties of candidates. Therefore, it is vital to carry out biophysical characterization to select the candidates with the lowest risks for manufacture. The antibody candidates AB1904Am3, AB1904Am5, AB1904Am6 and AB1904Am10 were analyzed by UV280 to detect protein concentration, by SEC-HPLC and reduced CE-SDS (rCE-SDS) and non-reduced CE-SDS (nrCE-SDS) to detect molecular size variants, by HIC-HPLC to evaluate hydrophobicity property, by DSF to detect thermal stability, and by iCIEF to detect pI value and charge variants. L0H0 was an in-house generated omalizumab biosimilar antibody. The comparability study including biophysical properties and biological activity was evaluated between L0H0 and omalizumab. Our results demonstrated that L0H0 had the high similarity both in biophysical characteristics and biological activities compared with original omalizumab antibody (**Supplementary Table 1**). In addition, the profiles of charge variants were highly similar between L0H0 and omalizumab. Altogether, the results of comparability study suggested that L0H0 had similar product quality, activity and pharmacokinetics as omalizumab. The yield of all antibody candidates was more than twice as many as that of L0H0 (**Figure 2B**). Higher expression level of antibody may lead to lower cost of manufacture. Purity evaluated by SEC-HPLC, nrCE-SDS and rCE-SDS and Fab Tm values evaluated by DSF as well as hydrophobicity evaluated by HIC-HPLC of all above antibody candidates were almost the same as L0H0. All antibody candidates’ pI values of their main peak were above 7.5 and slightly higher than L0H0 (**Figure 2C**). Charge variants profiles of all candidates indicated obvious difference compared with L0H0 **(****Figure 2G****)**. The main peak of AB1904Am5 was only 21%, and the acidic peak reached more than 69%, which was significantly lower than that of the L0H0 **(****Figures 2D, E****)**. The other three antibody candidates had a high proportion of the main peak purity, which was more than 80% **(****Figure 2D****)**, and the acid peak was slightly lower than that of L0H0 **(****Figure 2E****)**. The basic peaks showed no obvious difference among four candidates **(****Figure 2F****)**. Therefore, AB1904Am3 and AB1904Am6 as well as AB1904Am10 were chosen as the candidates for the following stability studies.

### Candidate Antibody Stability Profiling by Forced Degradation Studies

Forced degradation studies play a critical role in providing vital information to support therapeutic monoclonal antibody development and offer an opportunity to understand in-depth biophysical properties of candidates, including manufacturability evaluation, critical quality attributes confirmation, major degradation pathways, product variants identification, and predict the long-term stability (41). M252Y/S254T/T256E (YTE) mutation in Fc fragment was introduced into AB1904Am3, AB1904Am10 and AB1904Am6 to yield AB1904Am13, AB1904Am15 and AB1904Am16, respectively, for extending serum half-life. A series of stress conditions were applied, including high temperature (40°C), freeze-thaw, high pH (pH 9.0) and low pH (pH 5.0). Samples were taken at different time points and analyzed by SEC-HPLC, nrCE-SDS, rCE-SDS, iCIEF. Peptide mapping was applicated to quantify the formation of post-translational modifications. Among the stress conditions, stability profile of candidates at high temperature and low pH showed the most significant difference compared with L0H0.

**Figure 3:**
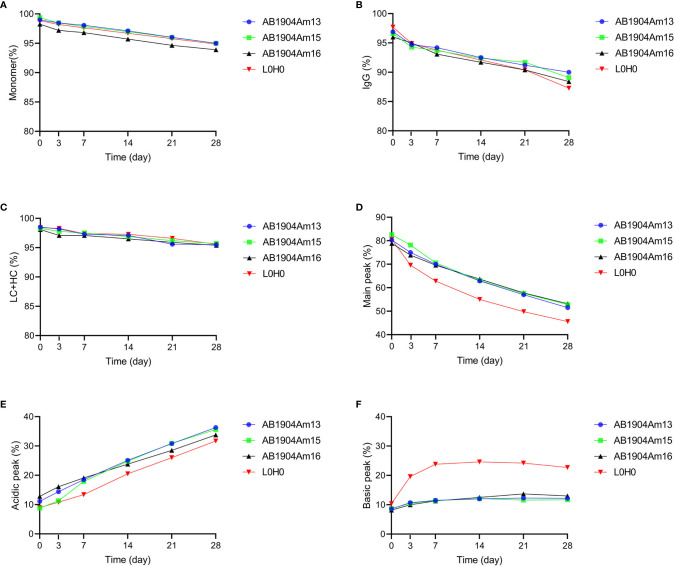
Stability analysis at 40°C over 28 days for antibody candidates AB1904Am13, AB1904Am15, and AB1904Am16, which were introduced YTE mutation into the Fc fragment of AB1904Am3, AB1904Am10 and AB1904Am6, respectively. **(A)** Percentage of monomer was measured by SEC-HPLC. **(B)** Percentage of total IgG purity was measured by nrCE-SDS. **(C)** Percentage of LC and HC was measured by rCE-SDS. Percentage of main peak **(D)**, acidic variants **(E)**, and basic variants **(F)** were measured by iCIEF.

As indicated in **Figures 3A–D**, the results obtained from the four analytical methods showed the same trend. The purities of all antibody candidates declined gradually with the prolongation of time at high temperature of 40°C. The result of SEC-HPLC revealed that AB1904Am16 was more incline to induce aggregates and its purity was constantly lower than other candidates and L0H0 (**Figure 3A**). The data of nrCE-SDS and rCE-SDS did not show any significant difference in purity among three molecules and L0H0 during 28 days (**Figures 3B, C****)**. As to the findings of iCIEF method, the main peak of L0H0 decreased more obviously than those of other candidates during 28 days **(****Figure 3D****)**. With the reduction of the main peak, the acidic peak of all four samples increased sharply from about 10% up to about 35%, while the basic peak of AB1904Am13, AB1904Am15 and AB1904Am16 remained stable at approximately 10% and the basic peak of L0H0 went up to about 25% in the 7^th^ day and then maintained at this level for two weeks before descending (**Figures 3E, F****)**. On the basis of above results, both AB1904Am13 and AB1904Am15 were more stable than L0H0 at high temperature of 40°C.

**Figure 4:**
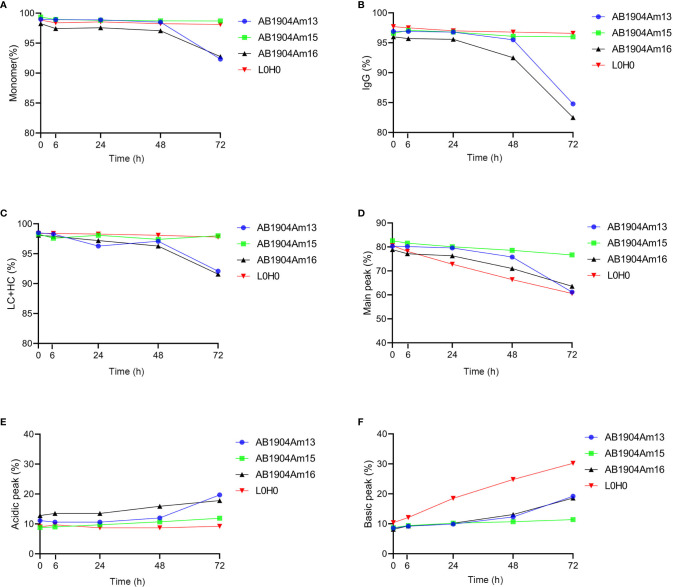
Stability analysis at low pH (pH 5.0/40°C) over 72 h for antibody candidates AB1904Am13, AB1904Am15 and AB1904Am16, which were introduced YTE mutation into the Fc fragment of AB1904Am3, AB1904Am10 and AB1904Am6, respectively. **(A)** Percentage of monomer was measured by SEC-HPLC. **(B)** Percentage of total IgG purity was measured by nrCE-SDS. **(C)** Percentage of LC and HC was measured by rCE-SDS. Percentage of main peak **(D)**, acidic variants **(E)**, and basic variants **(F)** were measured by iCIEF.

Under the forced degradation condition of low pH (pH 5.0/40°C), the results analyzed by SEC-HPLC, nrCE-SDS and rCE-SDS demonstrated that both AB1904Am15 and L0H0 were extremely stable during 72 h (**Figures 4A–C**). However, low pH condition induced more aggregates and fragments in AB1904Am13 and AB1904Am16 (**Figures 4A–C**). The information provided by iCIEF indicated the main peak of AB1904Am13, AB1904Am16 and L0H0 witnessed a downward trend, while AB1904Am15 kept relatively steady during 72 h (**Figure 4D**). After 24 h, the acidic peak of AB1904Am13, AB1904Am16 accumulated faster than AB1904Am15 and L0H0 (**Figure 4E**). It is noticeable that the percentage of the basic peak of L0H0 increased fast, from 10 up to above 30%, the basic peak of AB1904Am13 and AB1904Am16 increased slowly, while AB1904Am15 almost had no change (**Figure 4F**). The results of low pH treatment revealed that AB1904Am15 was the most stable candidate.

Taken together, AB1904Am15 showed better stability and less charge variants than AB1904Am13, AB1904Am16 and L0H0 under the stress conditions. AB1904Am13, AB1904Am16, and L0H0 were sensitive to temperature and pH treatment, and more aggregates, antibody fragments as well as charge variants were observed under stress conditions.

### Identification of PTMs by LC–MS/MS

**Figure 5:**
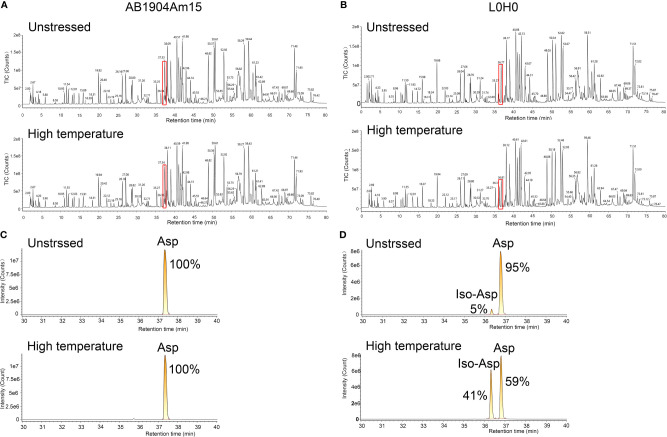
Peptide mapping analysis of AB1904Am15 and L0H0 by LC–MS/MS. Total ion chromatogram of the peptide map of AB1904Am15 **(A)** and L0H0 **(B)** at 40°C after 28 days. Highlighted area shows minor difference between fractions. Extracted ion chromatogram of CDR-L1 peptide of AB1904Am15 **(C)** and L0H0 **(D)** exhibiting a difference between the fractions at unstressed condition and high temperature (40°C) after 28 days. The elution peaks corresponding to DG and isoDG containing peptides were labeled as “Asp” and “IsoAsp”, respectively.

To identify the modifications causing the change of charge variants in the stress test, peptide mapping analysis by LC–MS/MS was carried out to investigate the relation between modifications and charge variants, especially basic charge variants. According to PTMs analysis results, we firstly found the modifications induced in stress conditions were asparagine deamidation which induced acidic variants and methionine oxidation which induced basic variants, while there was almost no difference in these modifications of all samples. However, there was significant difference of basic charge variants in AB1904Am15 and L0H0 at high temperature and low pH conditions, which may be induced by Asp isomerization. For Asp isomerization, it was difficult for MS/MS spectrum to differentiate an Asp from an iso-Asp because of no difference of molecular weight between originator and isomer. As shown in **Figures 5A, B**, the overview of peptide maps digested by trypsin showed no difference. Nevertheless, a deeper investigation revealed minor differences in the peptide mapping between 36 and 37 min. The difference in the peptide mapping was found to be associated with one of peptide sequences contained an Asp-Gly isomerization hotspot in the CDR-L1 of L0H0 (**Figure 5B**). An extracted ion chromatogram for this peptide was shown in **Figure 5D**, there were two peaks under both untreated and stress condition corresponding to this peptide in L0H0. The earlier eluting peak at 36.3 min was identified to be iso-Asp peptide because of more hydrophilic, which was increased from 5 to 41% in intensity under stressed conditions. Moreover, because of removing this isomerization hotspot in CDR-L1, there was only one peak corresponding to this peptide in the extracted ion chromatogram of AB1904Am15 (**Figure 5C**) under both untreated condition and stress condition. With the results of both charge variants by ICIEF and peptide mapping by LC–MS/MS, we found that the significant difference of basic peaks portion of L0H0 at stress condition was due to aspartic acid isomerization inducing in CDR-L1. According to the results of forced degradation studies, the stability of AB1904Am15 was superior to other candidates and control L0H0 and it was chosen as a lead candidate for the following study.

### Biological Characterization of AB1904Am15

**Figure 6:**
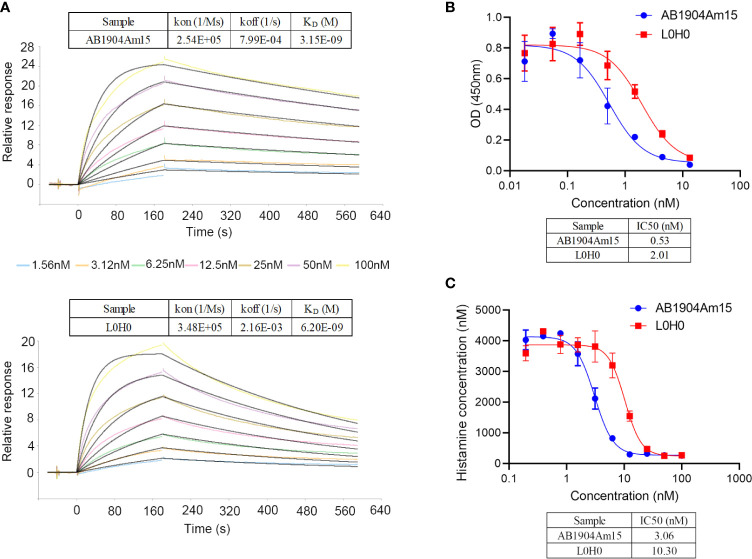
Biological characterization of AB1904Am15. **(A)** Binding affinity of AB1904Am15 and L0H0 to IgE was analyzed by SPR. **(B)** Blocking activity of AB1904Am15 and L0H0 to IgE receptor (FcϵRI) was measured by a competitive ELISA. **(C)** Histamine releasing assay by FcϵRI-expressing RBL-2H3 cells. Results were present as mean ± SD.

In this study, SPR results revealed that the affinity of AB1904Am15 to human IgE was approximately 2-fold higher than that of L0H0 (**Figure 6A****)**. The prime mechanism of action of anti-IgE antibody was to inhibit the IgE-mediated various allergic symptoms by inhibition of IgE interacting with FcϵRI on basophils and mast cells. Therefore, AB1904Am15 and L0H0 were assessed for their ability to prevent IgE binding to FcϵRI in protein-based receptor binding inhibition assay and cell-based functional assay. AB1904Am15 showed elevated potency in blocking the interaction of IgE with FcϵRI protein (**Figure 6B**) and inhibiting human IgE-induced histamine release in FcϵRI-expressing RBL-2H3 cells (**Figure 6C**), which were 3-fold higher than that of L0H0. In summary, AB1904Am15 showed higher affinity and elevated potency in blocking the interaction of human IgE with its primary receptor FcϵRI than that of L0H0 after antibody engineering.

### Pharmacokinetic Profile and *In Vivo* Efficacy of AB1905Am15

**Figure 7:**
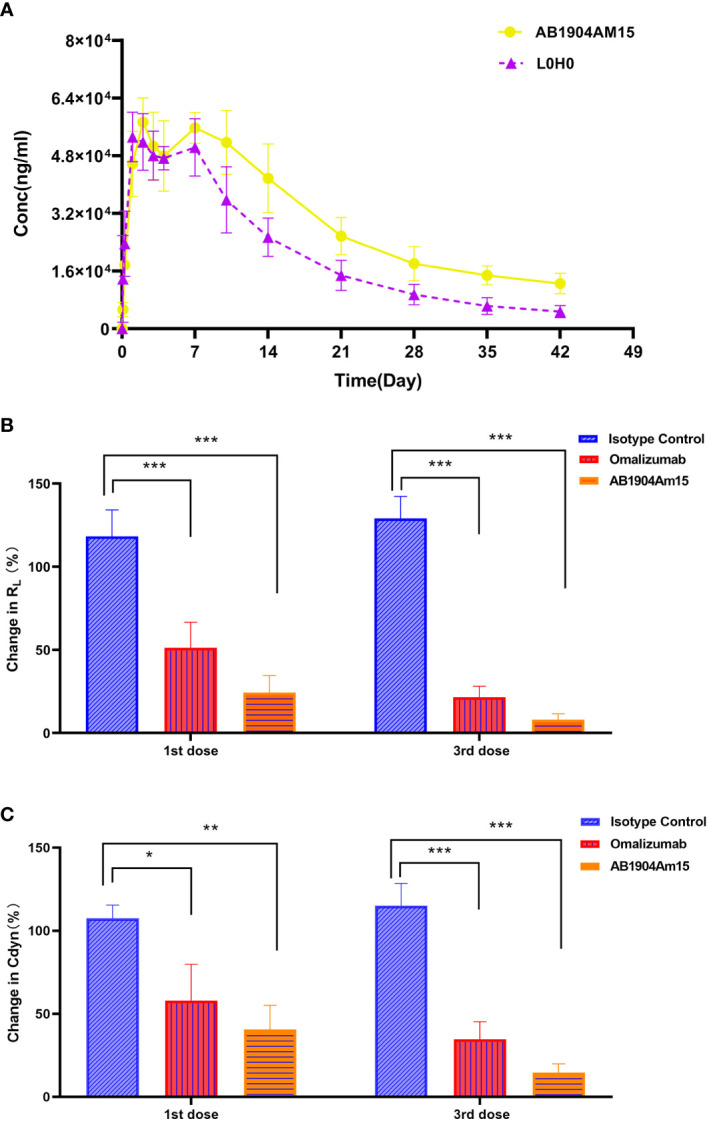
Pharmacokinetic and *in vivo* efficacy study of AB1904Am15. **(A)** Pharmacokinetic study of AB1904Am15 in hFcRn transgenic mouse. Group mean (± SD) serum antibody concentrations following single subcutaneous administration of 10.0 mg/kg of AB1904Am15 or L0H0 in hFcRn transgenic mouse. *In vivo* efficacy study of AB1904Am15 in cynomolgus monkey asthma model induced by DNP-As. Changes in R_L_ **(B)** and Cdyn **(C)** were evaluated after administration of 10.0 mg/kg of AB1904Am15 and omalizumab. Results were expressed as mean ± SD (n = 4, *P < 0.05, **P < 0.01, ***P < 0.001).

The PK profiles of AB1904Am15 and L0H0 following a single subcutaneous administration at 10 mg/kg in hFcRn transgenic mice were shown in **Figure 7A** and the PK parameters were summarized in **Table 1**. The half-life (t1/2) of L0H0 was about 12.9 days. AB1904Am15 with YTE mutations in the Fc fragment had a half-life (t1/2) of 35.1 days, which showed more than 2-fold increase compared with L0H0.

**Table 1:** Summary of pharmacokinetic parameters for AB1904Am15 and L0H0 in hFcRn transgenic mouse (mean ± SD).

Treatment	AB1904Am15 (n = 4)	L0H0 (n = 4)
t_1/2_ (day)	35.1 ± 26.7	12.9 ± 1.8
Tmax (day)	5.3 ± 3.9	2.8 ± 2.9
Cmax (μg/ml)	58.4 ± 6.6	53.7 ± 6.4
AUClast (day ∗ μg/ml)	1282.8 ± 228.4	898.7 ± 155.7
AUCINF_obs (day ∗ μg/ml)	1959.1 ± 719.1	989.2 ± 194.7

To verify the *in vivo* efficacy, AB1904Am15 were evaluated in a Cynomolgus monkey model of asthma induced by DNP-As allergen and omalizumab was used as a control. Either AB1904Am15 or omalizumab was administered subcutaneously at 10 mg/kg once a week. Lung airway resistance (R_L_) and dynamic lung compliance (Cdyn) were measured. As shown in **Figures 7B, C**, the monkeys treated with either AB1904Am15 or omalizumab showed significant changes in R_L_ and in Cdyn compared with the isotype control group at 24 h post 1st and 3rd dosing. Because of variability among monkeys, the P value of AB1904Am15 vs. omalizumab was slightly higher than 0.05. However, the mean values of change in R_L_ and Cdyn demonstrated that there was a sharp decline in change in R_L_ and Cdyn of the AB1904Am15 group when compared with omalizumab group, indicating that AB1904Am15 was more potent than omalizumab.

To further elucidate the underlying mechanism of *in vivo* efficacy optimization, AB1904Am15 (with YTE mutation in Fc region) and its wild type antibody (without YTE mutation in Fc region), which had same Fab regions, were included, and their suppression of free IgE were evaluated in cynomolgus monkeys compared with L0H0 to probe if the improved *in vivo* efficacy was resulted from the engineered Fab regions or YTE mutation in Fc regions, or both (**Supplementary Figure 2**). In the study, AB1904Am15 wild type antibody (without YTE mutation in Fc region) exhibited higher suppression of free IgE than L0H0, suggesting that the engineered Fab regions contributed to the elevated *in vivo* efficacy. In addition, AB1904Am15-induced suppression of free IgE was greater than its wild type antibody (without YTE mutation in Fc region), suggesting the YTE mutation also contributed to the improved efficacy. Collectively, the results demonstrated that the improved *in vivo* efficacy of AB1904Am15 was mediated by both engineered Fab regions and YTE mutation in Fc regions.

In conclusion, the results of the pharmacokinetics and *in vivo* efficacy suggested that AB1904Am15 had better pharmacokinetic profiles and efficacy than omalizumab, and its enhanced *in vivo* efficacy was resulted from the engineered Fab regions and YTE mutation in Fc regions.

## Discussion

The discovery of IgE and the identification of its key role in the pathogenesis of allergic disease have laid the foundation for the development of therapeutic anti-IgE approaches. Omalizumab, a humanized monoclonal antibody, was the only approved anti-IgE antibody on the market for clinical use till now. Since there are some limitations associated with omalizumab (28–31), a lot of efforts have been made to develop alternative anti-IgE antibodies that could potentially overcome these challenges and lead to better clinical outcomes (31).

Over the last decades, several anti-IgE antibodies have been generated and assessed in pre-clinical studies or clinical trials (31, 42–44). To date, besides ligelizumab (QGE031) is still under active investigation in clinical trials, other antibodies have been discontinued due to strategic decisions or failure to achieve primary endpoints in clinical trials (31, 43, 45). Ligelizumab is a novel humanized monoclonal antibody with high affinity to IgE. Because of different mechanistic and functional profile compared with omalizumab (45), ligelizumab lacked superior efficacy in phase II clinical trials with allergic asthma patients compared to omalizumab (31, 45). Therefore, ligelizumab was given up for the treatment of allergic asthma and chosen for the treatment of chronic spontaneous urticaria (CSU).

Based on the lessons learned from the previous studies (31, 42–44), it seems that too much emphasis has been placed on binding characteristics and the generation of novel mechanism of action different from omalizumab, and the less attention has been paid to fully understand or consider the exact mechanisms underlying omalizumab-dependent regulation of IgE production resulting in failure in clinical trials. Therefore, there certainly still exists a lot of challenges as well as room for improvement of anti-IgE biologicals. In this study, we attempted to improve anti-IgE therapeutics based on omalizumab for overcoming some limitations associated with omalizumab in stability and dosage restriction in clinical application. Because of following the modes-of-action of omalizumab, this study might increase chances of success in clinical trials and has the potential to lead to better clinical outcomes.

For stability, we removed two Asp isomerization hotspots in CDRs to improve its chemical stability. Meanwhile, we replaced several murine amino acids in the framework region of omalizumab with human source to reduce the potential risk of generating human anti-mouse antibody response. However, our assumption of minimizing the potential risk of immunogenicity was still needed to be verified by clinical trials in the future.

For limitation of clinical application, the clinical dosing of omalizumab was based on the body weight and serum total IgE level. Individuals with high body weight (>150 kg) or with higher free IgE levels (>700 IU/ml) may be excluded from dosing table or require frequent injections or high dosage (27, 30). Therefore, the development of an anti-IgE antibody candidate with higher affinity to IgE and longer half-life might provide the opportunity to reduce the dosage or dosing frequency and may offer the option to treat a broad patient population. Yeast display technology was applied for affinity maturation. AB1904Am3, AB1904Am6 and AB1904Am10 were chosen as candidates because of their higher affinity and desired biophysical properties. Extension of the antibody half-life can be achieved by Fc engineering. One of prototypical example of FcRn affinity enhancing Fc mutants is the M252Y/S254T/T256E(YTE) mutation (46, 47). In this study, YTE mutation in Fc fragment was applied to AB1904Am3, AB1904Am10 and AB1904Am6 to yield AB1904Am13, AB1904Am15, and AB1904Am16, respectively. All of them were tested under forced degradation conditions and evaluated for the subsequent change of biophysical properties. According to the results of forced degradation studies, AB1904Am15 showed greater stability compared with other candidates and L0H0 under stress conditions. The results of peptide mapping analysis by LC–MS/MS indicated that the basic charge variants accumulated under stress conditions were related to Asp isomerization in its CDR-L1. Previous studies also found that the variants resulting from the isomerization, particularly if the residues involved in the CDRs of antibody, brought challenges to efficacy, antibody manufacturing, and quality control (28, 29, 34). Therefore, AB1904Am15 was selected as a lead candidate for further study because of greater stability.

Moreover, AB1904Am15 showed higher affinity to IgE than L0H0, and demonstrated a correspondingly enhanced inhibition of IgE binding to FcϵRI and enhanced suppression of IgE induced histamine release in FcϵRI-expressing RBL-2H3 cells compared with L0H0. Previous studies have reported that superior IgE suppression may translate to better clinical outcomes (27, 42, 45). The pharmacokinetic study of anti-IgE antibodies in human FcRn transgenic mouse model indicated the serum half-life of AB1904Am15 was extended more than 2-fold compared with L0H0, which was consistent with the previous reports (48, 49). Robbie GJ and Griffin MP found YTE mutation in nirsevimab extended serum half-life more than 2- to 4-fold compared with its wild type antibody (motavizumab) in clinical trials (48, 49). Best of all, *in vivo* efficacy study demonstrated that AB1904Am15 was more potent than omalizumab. To our current knowledge, this is the first reported study showing the *in vivo* efficacy of anti-IgE antibody in cynomolgus monkey asthma model. To elucidate whether the improved *in vivo* efficacy was mediated by either the engineered Fab regions or YTE mutation in Fc regions, or both, the suppression of free IgE induced by AB1904Am15 (with YTE mutation in Fc region) and its wild type antibody (without YTE mutation in Fc region) was evaluated in cynomolgus monkeys compared with L0H0. The results indicated that both the engineered Fab regions and YTE mutation in Fc regions contributed to the enhanced *in vivo* efficacy of AB1904Am15. Enhanced *in vitro* biological activities and *in vivo* efficacy as well as prolonged serum half-life may bring about the following clinical benefits: lower volume of injection required, lower doses required to achieve clinical benefits, decreased frequency of drug administration.

Previous studies have reported that omalizumab is effective in treating asthma patients with chronic obstructive pulmonary disease (COPD) overlap syndrome or with cardiovascular complications by decreasing coagulant proteins (D-Dimer) and proinflammatory cytokine levels (50, 51). In consideration of these facts, Yalcin AD and Yalcin AN highlighted that omalizumab therapy for patients with severe COVID-19 might contribute to good clinical outcomes (52). AB1904Am15, a novel anti-IgE antibody, might have the potential in treating the asthma patients with COPD overlap syndrome and cardiovascular diseases, and protecting the patients with severe COVID-19 and leading to greater survival.

**Figure 8:**
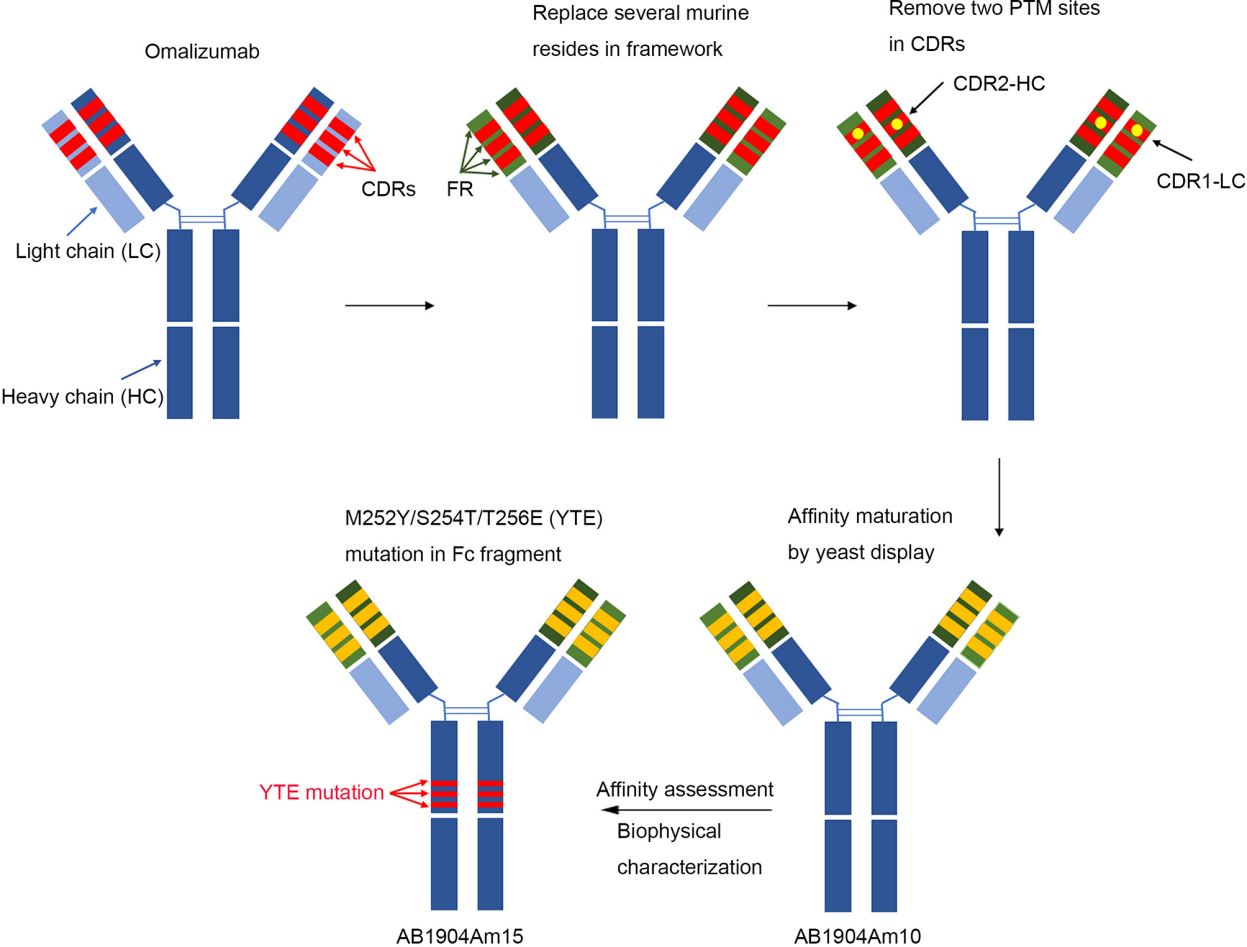
Schematic illustration of generation of AB1904Am15. In brief, antibody engineering approaches were applied to replace several murine amino acids with human source in the framework region of omalizumab and remove two aspartic acid isomerization hotspots in CDRs of omalizumab. Then, affinity mature was carried out by yeast display platform using above optimized sequence as the template, and AB1904Am10 were acquired by affinity evaluation and biophysical characterization. Finally, M252Y/S254T/T256E (YTE) mutation in the crystallizable fragment was introduced into AB1904Am10 to yield AB1904Am15.

In summary, an integrated antibody engineering approach was applied to generate AB1904Am15 (**Figure 8**) for overcoming some limitations associated with omalizumab. AB1904Am15 showed desired biophysical properties, improved stability, enhanced efficacy *in vitro* and *in vivo*, and prolonged serum half-life in comparison with omalizumab. Our preclinical studies suggested that AB1904Am15 might be a more potent anti-IgE monoclonal antibody for the treatment of allergic diseases in the future.

## Data Availability Statement

The original contributions presented in the study are included in the article/**Supplementary Materials**, further inquiries can be directed to the corresponding authors.

## Ethics Statement

The animal study was reviewed and approved by the Institutional Animal Care and Use Committee.

## Author Contributions

SD designed and directed the project. DJ, HG, and XW provided technical guidance and reviewed the manuscript. ZP designed the *in vivo* efficacy study. XL analyzed the sequencing data. CG carried out the affinity maturation. XC performed the *in vitro* activity evaluation. JZ performed the PK analysis. ZX assisted in the data analysis. PL carried out the biophysical analysis, stability study, processed the experimental data, drafted the manuscript, and designed the figures. All authors contributed to the article and approved the submitted version.

## Conflict of Interest

PL, ZP, CG, XC, XL, JZ, ZX, XW, HG, and S-JD were employed by Shanghai Jemincare Pharmaceuticals Co., Ltd.

The remaining author declares that the research was conducted in the absence of any commercial or financial relationships that could be construed as a potential conflict of interest.
