# Supplementary material for: An Omalizumab Biobetter Antibody With Improved Stability and Efficacy for the Treatment of Allergic Diseases
Source: Front Immunol. 2020 Nov 27;11:596908. doi: 10.3389/fimmu.2020.596908 (PMC7728613; doi:10.3389/fimmu.2020.596908)
Supplement: Supplementary file 1 [file DataSheet_1.docx]

**Supplementary Material**

**Supplementary Figure 1.** The mutation sites in the VH and VL regions compared with omalizumab (*represent mutation sites).

**Supplementary Table 1.** Summary of biophysical and biological comparability assessment between L0H0 and omalizumab.

**Supplementary Figure 2.** Free IgE suppression in cynomolgus monkeys.

**
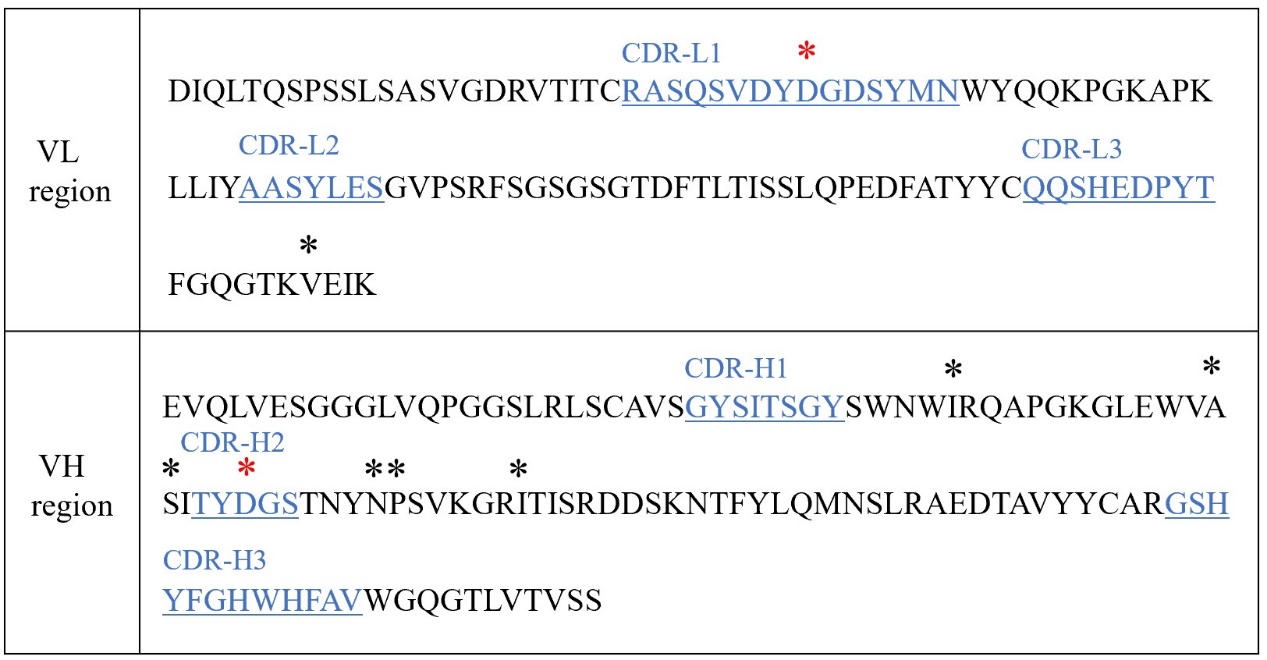
Supplementary Figure 1.** The mutation sites in the VH and VL regions compared with omalizumab (*represent mutation sites). Compared with the sequence of omalizumab, two amino acids in the CDRs of omalizumab were removed to improve antibody stability (*in red), Meanwhile, seven murine amino acids in the framework region of omalizumab were replaced with human source to minimize the potential immunogenicity (*in black).

**Supplementary Table 1. Summary of biophysical and biological comparability assessment between L0H0 and omalizumab**

| Category | Assay | | Sample name | |
| --- | --- | --- | --- | --- |
|  |  |  | L0H0 | omalizumab |
| Biophysical characteristics | SEC-HPLC | Monomer (%) | 99.7 | 99.6 |
|  | nrCE-SDS | IgG (%) | 97.7 | 97.6 |
|  | rCE-SDS | LC+HC (%) | 98.4 | 98.2 |
|  | DSF | Fab Tm (℃) | 88.5 | 88.0 |
|  | iCIEF | pI value | 7.5 | 7.5 |
|  | Charge variants assay by iCIEF | Acidic peaks (%) | 10.6 | 12.2 |
|  |  | Main peak (%) | 80.6 | 79.3 |
|  |  | Basic peaks (%) | 8.9 | 8.5 |
| Biological activity | Affinity to IgE (nM) | | 0.40 | 0.47 |
|  | Blocking activity (nM) | | 1.85 | 1.59 |
|  | Affinity to FcRn (μM) | | 0.09 | 0.10 |

**
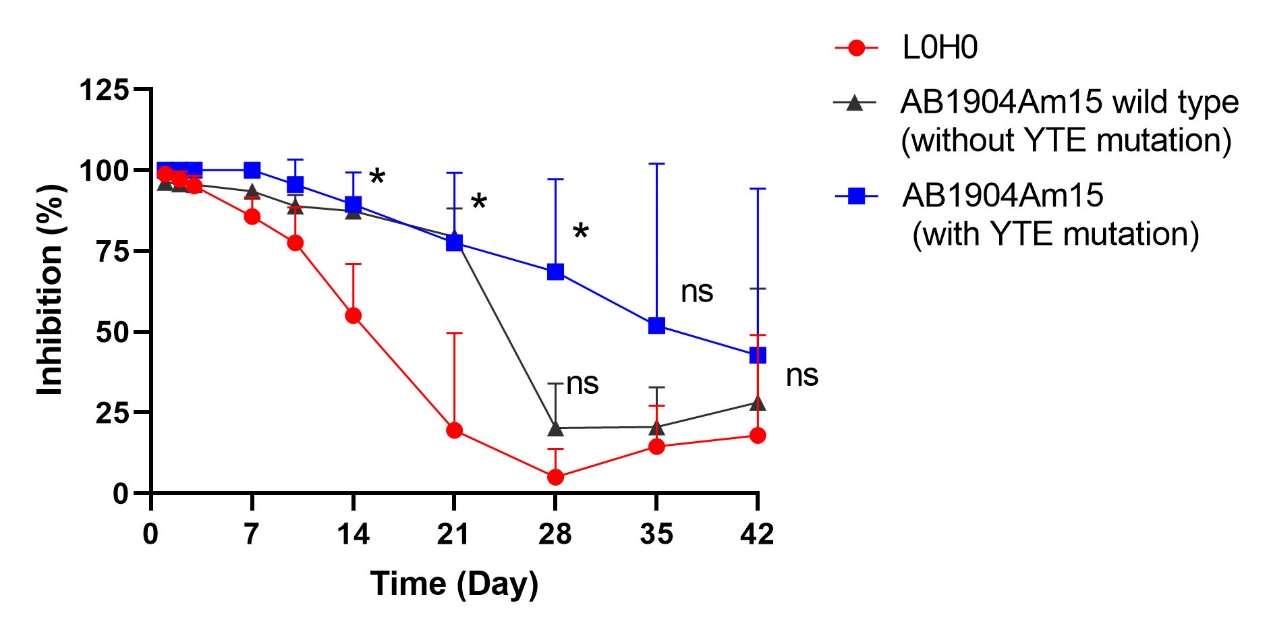
****Supplementary Figure 2. Free IgE suppression in cynomolgus monkeys.** Cynomolgus monkeys received a single dose of 10 mg/kg test antibody via subcutaneous administration (n=3/group). Serum samples were collected at the following time points: pre-dose, 1,2, 3, 7, 10, 14, 21, 28, 35, and 42 days post dose. Serum concentrations of free IgE were measured by an ELISA method. Results were presented as mean ± SD (**P < 0.05*, ns, no significance, performed by *t*-test**)**.
